# Wild Species from the Asteraceae Family, Traditionally Consumed in Some Mediterranean Countries

**DOI:** 10.3390/plants14132006

**Published:** 2025-06-30

**Authors:** Ekaterina Kozuharova, Giuseppe Antonio Malfa, Rosaria Acquaviva, Benito Valdés, Daniela Batovska, Christina Stoycheva, Moh Rejdali, Pasquale Marino, Vivienne Spadaro

**Affiliations:** 1 Faculty of Pharmacy, Department of Pharmacognosy, Medical University-Sofia, 1000 Sofia, Bulgaria; christina9828@gmail.com; 2 Department of Drug and Health Sciences, University of Catania, Viale A. Doria 6, 95125 Catania, Italy; gmalfa@unict.it (G.A.M.); racquavi@unict.it (R.A.); 3 Research Centre on Nutraceuticals and Health Products (CERNUT), University of Catania, Viale A. Doria 6, 95125 Catania, Italy; 4 Department of Plant Biology and Ecology, University of Seville, Avda. Reina Mercedes s/n, 41012 Seville, Spain; bvaldes@us.es; 5 Institute of Chemical Engineering, Bulgarian Academy of Sciences, Acad. Georgi Bonchev Str., Bl. 103, 1113 Sofia, Bulgaria; danielabatovska@gmail.com; 6 Institut Agronomique et Vétérinaire Hassan II Rabat Maroc, Rabat 10112, Morocco; m_rejdali@hotmail.com; 7 PLANTA/Center for Research, Documentation and Training, Via Serraglio Vecchio 28, 90123 Palermo, Italy; marino@centroplantapalermo.org; 8 Department of Biological, Chemical and Pharmaceutical Sciences and Technologies, Section of Botany, Anthropology and Zoology, University of Palermo, Via Archirafi 38, 90123 Palermo, Italy; vivienne.spadaro@unipa.it

**Keywords:** edible wild plants, Compositae, ethnobotanical knowledge, similarity, distribution, Jaccard index, Mediterranean basin

## Abstract

Mediterranean countries represent a dynamic hub of cultural exchange, where wild plants play a significant role in culinary traditions. A substantial number of these plants belong to the Asteraceae family. The climate similarities across the region contribute to the common distribution ranges of the plants. While many species are widely distributed, others are confined to specific subregions, such as the western Mediterranean, eastern Mediterranean, or North Africa. Only six taxa of the traditionally consumed wild Asteraceae plants are endemic to just one country. This review focuses on wild plants from the Asteraceae family traditionally used as food across 13 study sites, comprising 11 countries in the Mediterranean and adjacent territories, including both mainland areas and three islands. The objective is to identify and analyze patterns of native distribution in relation to actual consumption. As a result, 167 edible wild plants from the Asteraceae family were identified. Their patterns of distribution and consumption are described and analyzed. The highest number of these edible wild plants from the Asteraceae family is consumed in Spain (*n* = 65), followed by southern Italy (*n* = 44) and Morocco (*n* = 32). A similar pattern of consumption is seen in Turkey (*n* = 24), Sicily (*n* = 23), Jordan and Palestine (*n* = 21), and Bulgaria (*n* = 21). It is notable that 106 plants are used as food in one particular country only, although most of them are distributed in several other countries. Many of the species consumed in certain countries are not used by neighboring populations, highlighting a limited cross-border transmission of ethnobotanical knowledge. The findings from a Jaccard index statistical analysis are discussed.

## 1. Introduction

The consumption of edible wild plants is deeply rooted in the cultural heritage of various regions, reflecting the local traditions and identities of their communities. The Mediterranean basin, in particular, stands out as a melting pot of diverse cultures and religious traditions, shaped over centuries by significant migrations, invasions, and conquests. For example, during the 10th and 11th centuries, various European territories were taken over by Muslim forces [1]. Later, in the 14th and 15th centuries, the Ottoman Empire expanded its reach, moving west into the Balkans, south into the Near East and North Africa, and east toward the Caspian Sea, encompassing regions like Armenia [2]. Additionally, historical ties between eastern Spain and Sicily can be traced back to the 13th century when Sicily was under the rule of the Kingdom of Aragon. The island also managed to maintain its political stability by resisting pressures from dominant maritime republics like Pisa and Genoa [3]. Furthermore, notable cultural exchanges occurred between Spain and Morocco during the Middle Ages [4].

Trade routes crisscrossing the Mediterranean Sea played a crucial role in the transmission of knowledge among the diverse cultures inhabiting the region, including practices related to the consumption of wild plants as food. Due to enduring Mediterranean customs, many of these wild plants remain integral to local cuisines today [5]. Since ancient times, native plant species have served as one of the most accessible food sources. Across the globe, communities have explored and consumed various parts of the plants available in their immediate environments. Over time, numerous plant species underwent prolonged selection and domestication, and as human populations relocated to new areas, they brought these plants along with them [6]. This has given rise to a rich tapestry of culinary traditions; however, while they often share similarities, each region has also preserved its own unique food customs.

Since ancient times, wild plants consumed by populations in the Mediterranean have attracted attention due to their positive effects on human health. The notable life expectancy of people in this region is often linked to their dietary habits. The Mediterranean diet, in particular, is widely regarded as a health-promoting and environmentally sustainable model. It supports biodiversity, fosters a deep cultural identity, and helps preserve long-standing traditions among communities throughout the Mediterranean basin [7]. Typically, the traditional diet in these countries emphasizes the consumption of a wide variety of foraged plant foods. These include climbing plants with tender shoots, wild tubers, leafy greens, fruits from native trees and shrubs, and aromatic herbs used for seasoning [8,9]. In recent years, growing scientific interest has focused on edible wild plants, especially regarding the health-related properties of their phytochemicals. This has led to them being referred to as “new functional foods” [10].

In the Mediterranean region, approximately 2300 species of fungi and plants are foraged from the wild for food, with a significant proportion belonging to the Asteraceae family. For instance, in Andalusia, 336 wild plant taxa—representing about 7% of the region’s total wild flora—are gathered and consumed, and Asteraceae members lead the list with 65 species (18% of the plants used for food) [11]. The Asteraceae family includes over 1600 genera and 25,000 species worldwide. This plant family has long been valued for its diverse usesok, ranging from culinary and traditional medicinal applications in ancient times to modern roles in the pharmaceutical and cosmetic industries [12,13].

The shared characteristics of the Mediterranean climate influence the distribution patterns of many plant species across the region. While numerous plants are widespread throughout the entire area, some are found only in either the western or eastern parts of the Mediterranean. Certain species are exclusive to North Africa, whereas others do not extend as far south [14]. As a result, these floristic differences shape the use of particular wild plants as food, making them common in some countries or regions, yet absent from the traditional diets of others.

**Figure 1:**
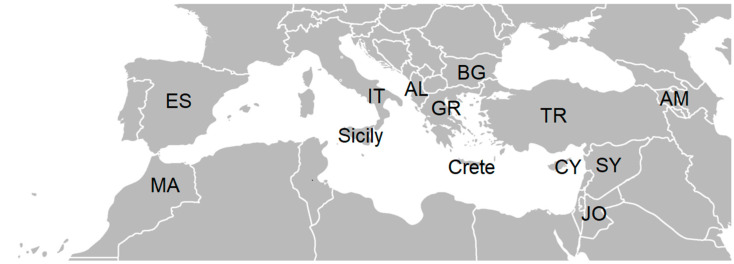
Study sites. Legend: JO—Jordan and Palestine; IT—southern Italy; MA—Morocco; Sicily; ES—Spain; SY—Syria; TR—Turkey (Türkiye); AL—Albania; AM—Armenia; BG—Bulgaria; Crete; CY—Cyprus; GR—Greece. Credit https://commons.wikimedia.org/wiki/File:BlankMap-Europe-v4.png. accessed on 16 May 2020.

The aim of this review is to study wild plants from the Asteraceae family that are traditionally consumed in 13 study sites across 11 countries, including three islands in the Mediterranean, and their adjacent territories (Figure 1). Here we endeavor to trace the native distribution and patterns of cultural exchange. We aim to test the following statements: (1) the neighboring countries have high similarity in the distribution of Asteraceae taxa, and (2) the countries with a common history have high similarity in consumption patterns.

## 2. Results

### 2.1. Distribution of Wild Asteraceae Species, Traditionally Used as Food

Based on our literature review, we compiled a list of 167 wild Asteraceae species traditionally consumed across 13 study locations, including mainland areas and three islands, spanning 11 countries within the Mediterranean region and its neighboring territories (see Figure 1, Table 1).

The distribution of these 167 taxa is either all over the territory of the studied sites or restricted to certain parts (Table 1).

**Figure 2:**
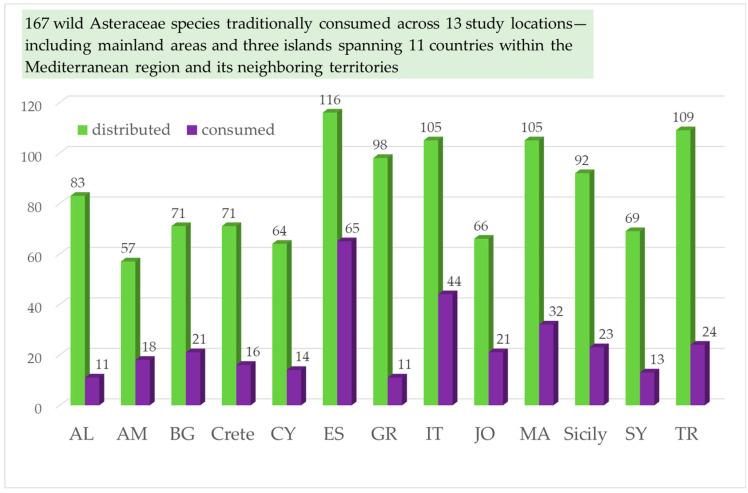
Number of wild species from the Asteraceae family distributed and consumed in each of the study sites. The x-axis presents the studied countries, and the y-axis presents the number of wild species from the family Asteraceae, respectively distributed (green) and consumed (purple) in each of the study sites.

The listed 167 wild plants from family Asteraceae, consumed traditionally (Table 1) are documented from west to east as follows: 32 taxa in Morocco [15,16,17,18,19,20,21,22], 65 taxa in Spain [23,24,25], 23 taxa in Sicily [26,27,28,29], 44 taxa in southern Italy [30,31,32,33,34], 11 taxa in Albania [32,34,35,36], 11 taxa in Greece [37,38,39], 16 taxa in Crete [37,40,41], 14 taxa in Cyprus [37,41], 24 taxa in Turkey (Türkiye) [42,43,44,45,46], 21 taxa in Bulgaria [43,47,48,49,50,51], 18 taxa in Armenia [52,53,54,55,56,57,58,59], 13 taxa in Syria [60], 22 taxa in Jordan and Palestine [61,62,63,64] (Figure 2). The highest number of these edible wild plants from the Asteraceae family is consumed in Spain (*n* = 65), followed by southern Italy (*n* = 44) and Morocco (*n* = 32) (Figure 2). A similar pattern of consumption is seen in Turkey (*n* = 24), Sicily (*n* = 23), Jordan and Palestine (*n* = 21), and Bulgaria (*n* = 21) (Figure 2). This rating pattern may mean that the tradition of consumption is strongest in these countries. However, it may be a reflection of the depth of ethnobotanical studies in these countries compared to the others. 

**Figure 3:**
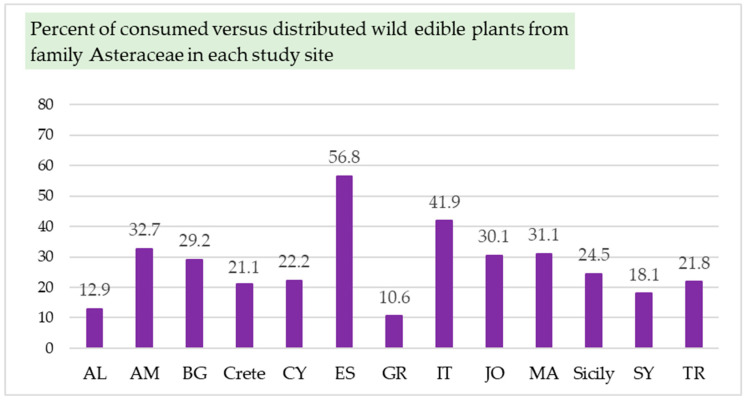
Percentage of traditionally consumed versus distributed edible wild plants from the Asteraceae family in each country. The x-axis presents the studied countries, and the y-axis presents the percentage of consumed versus distributed edible wild plants from the Asteraceae family in each country.

**Figure 4:**
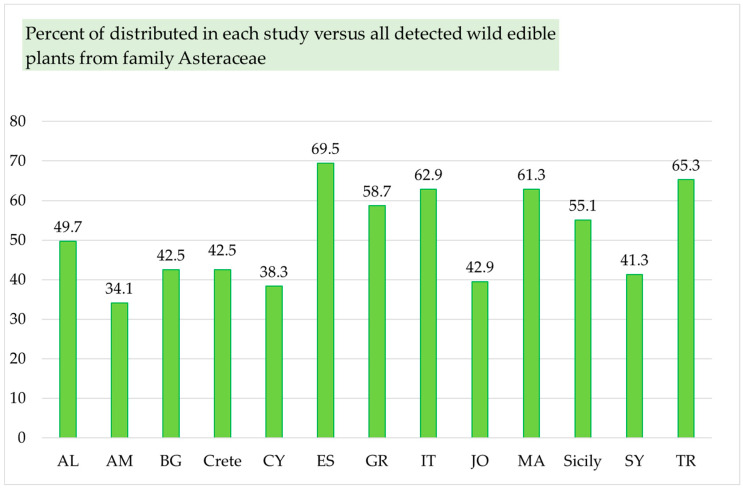
Percentage of distribution in each study site versus all detected edible wild plants from the Asteraceae family. The x-axis presents the studied countries, and the y-axis presents the percentage of distribution in each country versus all detected edible wild plants from the family Asteraceae.

It is also demonstrated that only a little more than half of the distributed edible plants from the Asteraceae family (in Spain), or often less than half (in the other countries), are traditionally consumed (Figure 3). Spain has the highest rate of utilization of edible wild Asteraceae species found within its territory (56.8%), followed by Italy (41.9%), Armenia (32.7%), Morocco (31.1%), Jordan and Palestine (30.1%), and Bulgaria (29.2%). The lowest level of consumption is recorded in Greece, where only 10.6% (11 taxa) of the edible wild Asteraceae members distributed within its borders are utilized (Figure 3). Notably, Spain hosts the largest number of wild plants recognized as edible from the Asteraceae family (116 taxa, or 69.5%. Figure 2 and Figure 4). Next is Turkey with 109 taxa, or 65.3%. Similarly, a high number of edible wild Asteraceae members grow in Italy (105 taxa or 62.9%), Morocco (105 taxa or 62.9%) and Greece (98 taxa or 58.7%, Figure 2 and Figure 4).

In general, the edible wild plants from the Asteraceae family have a wide distribution range, growing in many of the study sites. For example, in all the study sites, *Bellis perennis* L., *Calendula arvensis* (Vaill.) L. (although in Bulgaria it is only along the Black Sea coast), *Centaurea solstitialis* L., *Chondrilla juncea* L., *Cichorium intybus* L., *Cirsium vulgare* (Savi) Ten., *Dittrichia graveolens* (L.) Greuter, *Dittrichia viscosa* (L.) Greuter, *Lactuca serriola* L., *L. viminea* (L.) J. Presl & C. Presl, *Senecio vulgaris* L., *Silybum marianum* (L.) Gaertn., *Sonchus asper* (L.) Hill, *Sonchus oleraceus* L., *Tragopogon porrifolius* L., and *Urospermum picroides* (L.) F. W. Schmidt (Table 1).

Only a few of the traditionally consumed wild Asteraceae plants are endemic to just one country (Table 1), such as *Carduncellus dianius* Webb (Syn. *Carthamus dianius* (Webb) Coincy) and *Sonchus crassifolius* Willd. to Spain; *Sonchus pinnatifidus* Cav. to Morocco; *Crepis apula* (Fiori) Babc. to Italy; *Onopordum cyprium* Eig to Cyprus.

Several taxa have restricted distribution in several neighboring countries (Table 1).

The range of several taxa is restricted to the western Mediterranean, namely *Helminthotheca comosa* (Boiss.) Holub (Syn. *Picris comosa* (Boiss.) B. D. Jacks. to Spain, Morocco (and Algeria); *Carduus tenuiflorus* Curtis to Spain, Morocco (also France, Portugal, Algeria and several other countries); *Cynara humilis* L. to Spain, Morocco (also Portugal and Algeria); *C. tournefortii* Boiss. & Reut. to Spain, Morocco (also Portugal); *Pseudopodospermum crispatulum* (DC.) Zaika, Sukhor. & N. Kilian (Syn. *Scorzonera crispatula* (DC.) Boiss.) to Spain, Morocco (Portugal and Algeria); *Onopordum acaulon* L. to Spain, Morocco (as well as Portugal, Algeria and Sardinia); *O. corymbosum* Willk. to Spain (and France); *O. macracanthum* Schousb. to Spain, Morocco (also Baleares, Portugal, Algeria and Tunisia); *O. nervosum* Boiss. to Spain (and Portugal); *Scorzonera angustifolia* L. to Spain and Morocco (and Portugal); *S. schweinfurthii* Boiss. to Jordan (and Egypt), (Table 1, [14]).

Restricted to the eastern Mediterranean are *Arctium lappa* subsp. *platylepis* (Boiss. & Balansa) Arènes to Turkey (as well as Azerbaijan and Georgia); *Cynara syriaca* Boiss. to Syria, Jordan and Palestine (as well as Israel and Lebanon); *Echinops orientalis* Trautv. to Armenia and Turkey (as well as Azerbaijan, Nakhichevan, and North Caucasus); *Helichrysum rubicundum* (K. Koch) Bornm. to Armenia and Turkey (as well as Azerbaijan and Georgia); *Taraxacum cyprium* H. Lindb. to Cyprus, Jordan and Palestine (as well as Israel, and Lebanon); *Tragopogon reticulatus* Boiss. & A. Huet to Armenia and Turkey (as well as Azerbaijan and Georgia), (Table 1, [14]).

*Artemisia atlantica* Coss. & Durieu, *Chrysanthoglossum trifurcatum* (Desf.) B. H., *Otoglyphis pubescens* (Desf.) Pomel (Syn. *Aaronsohnia pubescens* (Desf.) K. Bremer & Humphries); and *Endopappus macrocarpus* Sch. Bip.; *Raponticum acaule* (L.) DC. grow only in North Africa (Morocco and neighboring countries) (Table 1, [14]).

**Figure 5:**
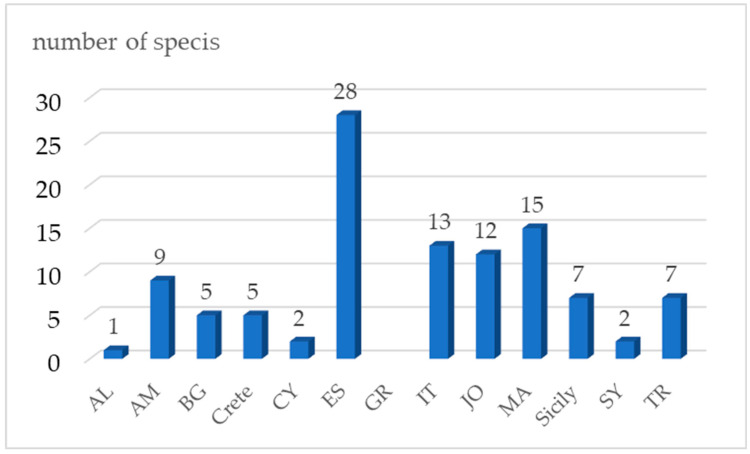
Number of wild plants from the Asteraceae family with limited consumption in only one country. The x-axis presents the studied countries, and the y-axis presents the number of wild species of the Asteraceae family traditionally consumed as food.

It is notable that 106 plants are used as food only in one particular country, although most are distributed in several other countries (Table 1). They represent 63.1% of all edible wild Asteraceae members in the studied sites. Such unique consumption is recorded in 12 study sites (all study sites except Greece). The specifics are as follows: 28 taxa are used exclusively in Spain (accounting for 43.1% of all edible wild plants from Asteraceae consumed there). Furthermore, 15 taxa are used as food only in Morocco (28.8% of all edible wild plants from Asteraceae consumed there), 13 taxa in southern Italy (29.5%), 12 taxa in Jordan and Palestine (54.5%), 9 taxa in Armenia (50%), 7 taxa in Turkey (29.2%), 7 taxa in Sicily (30.4%), 5 taxa in Bulgaria, (23.8) 5 taxa in Crete (31.3%), 2 taxa in Syria (15.4%), 2 taxa in Cyprus (14.2%) and 1 species in Albania (9.1%), (Figure 5).

### 2.2. Statistical Analyses of the Distribution and Consumption of Wild Umbellifers

#### Jaccard Index

The Jaccard index (JI) revealed notable differences in the level of similarity between country pairs in terms of traditional consumption of wild Asteraceae species, compared to the distribution pattern of these edible wild plants among the same country pairs.

**Figure 6:**
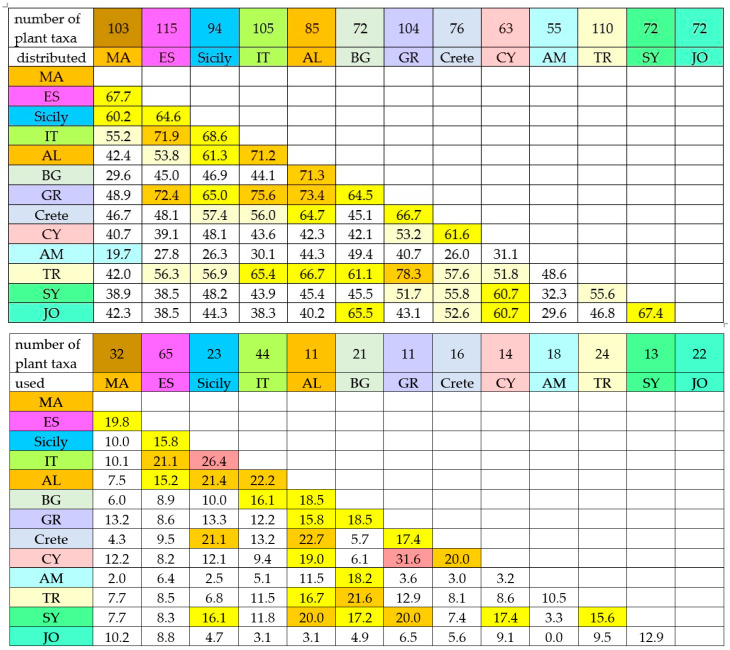
Similarity between pairs of countries in both the traditional consumption of wild Asteraceae and the distribution of these edible wild plants, expressed as JI%.

The similarity of distribution patterns (floristic similarity) between countries is rather high. Neighboring countries have higher similarity, although some distantly located study sites, such as the pairs Spain and Turkey or Spain and Albania, and several others, still have more than 50% similarity (Figure 6). This reflects the fact that the edible wild Asteraceae plants have a wide distribution. The highest similarity in terms of distribution patterns of edible wild plants from the Asteraceae family is observed between Greece and Turkey (JI = 78.3%), followed closely by Italy and Greece (JI = 75.6%), Albania and Greece (JI = 73.4%), Spain and Greece (JI = 72.4%), Spain and Italy (JI = 71.9%), Albania and Bulgaria (JI = 71.3%), Albania and Italy (JI = 71.2%). Seventeen pairs of countries have a similarity between 60 and 70% (Figure 6). Also, 13 pairs of countries have a similarity between 50 and 60% (Figure 6). Only one pair of countries has less than 20% similarity in the distribution of edible wild plants from the Asteraceae family, namely Morocco and Armenia (Figure 6).

However, pairwise similarity in consumption patterns (consumption similarity) diverges from the distribution patterns. In general, the JI values for consumption are much lower across all country pairs compared to the distribution patterns. Interestingly, the similarity between the consumption pairs does not follow the distribution patterns. Contrary to expectations, the pairs with the highest distribution similarity demonstrate low consumption similarity. For example, the pair Greece and Turkey with the highest distribution similarity (JI = 78.3%) has a low consumption similarity (JI = 12.9%). Also, the consumption similarity between Italy and Greece is JI = 12.2%, while the distribution similarity is the second highest (JI = 75.6%). Additionally, other pairs with high distribution similarity have low consumption similarity, namely Albania and Greece (JI = 73.4%) vs. (JI = 15.8%), Spain and Greece (JI = 72.4%) vs. (JI = 8.6%). Only several pairs with high distribution similarity have comparatively high consumption similarity and are as follows: Spain and Italy (JI = 71.9%) vs. (JI = 21.1%), Albania and Bulgaria (JI = 71.3%) vs. (18.5) and Albania and Italy (JI = 71.2%) vs. (JI = 22.2%).

The highest consumption similarity is recorded between Greece and Cyprus (JI = 31.6%), but their distribution similarity is moderate (53.2%). The second highest consumption similarity is recorded between southern Italy and Sicily (JI = 26.4%), with a high distribution similarity (68.6%), followed by Albania and Crete (JI = 22.7%), also with a high distribution similarity (64.7%) (Figure 6).

Contrary to expectations, the similarity in use patterns between Bulgaria and Turkey—two neighboring countries with shared historical ties under the Ottoman Empire—is not very high (JI = 21.6%). This is valid for Armenia and Turkey, too (JI = 10.5%). Similarly, the similarity in use patterns between Morocco and Spain (JI = 19.8%) is also lower than expected (Figure 6). A possible explanation is that the distribution similarity of the edible wild members of the Asteraceae family is moderate (Figure 6). But also, it suggests that ethnobotanical knowledge does not cross national borders as extensively as expected, since many plants used in one country are not utilized by neighboring countries. Of course, there are limitations to the interpretations due to the inconsistency of ethnobotanical data collection.

If we compare the floristic similarity with the consumption similarity of the pairs of studied countries, some interesting patterns are noticed. For example, the consumption similarity between Greece and Turkey (JI = 12.9%, Figure 6) is low, despite the high floristic similarity (JI = 78.3%, Figure 6) and common history [2]. The floristic similarity (JI) exceeds by a factor of about six times the consumption similarity (JI). When we compare Armenia and Turkey (Figure 6), the floristic similarity (JI = 48.6%) exceeds the consumption similarity (JI = 10.5%) by a factor of only 4.6. The consumption similarity between Bulgaria and Turkey (JI = 21.6%, Figure 6) is less than three times lower than the floristic similarity (JI = 61.1%, Figure 6). The common history of these three countries [2] influenced the culinary cultural exchange differently. The floristic similarity of Sicily and southern Italy (JI = 68.6%) exceeds their consumption similarity by a factor of only two (JI = 26.4%). Such is also the case with Sicily and Albania, as well as Albania and Cyprus (Figure 6). Here, obviously, the cultural exchange is more efficient. Interestingly, the floristic similarity of pairs of countries like Jordan and Palestine and Bulgaria exceeds the consumption similarity by a factor of more than 13. Jordan and Palestine and Albania are a similar case. Also, the similarity of consumption between this Middle East region and Italy is 12.4 times lower than the floristic similarity regarding edible plants from the Asteraceae family. This is not surprising considering the fact that most plants from this family identified as edible have a wide distribution on the one hand (Table 1), and on the other hand, the countries are some distance apart (Figure 1). This notable distance obviously has restricted wild plants’ cultural culinary exchange, even though, during the Crusades, the Balkan Peninsula was a crossroad [1]. Surprisingly, when comparing the floristic similarity with consumption similarity of Syria to those of Albania, Bulgaria and Greece, the ratio is less than three times, and we cannot find a convincing explanation for this result. Although Turkey and Syria are neighboring countries with a common border (Figure 1), neither the floristic similarity nor the consumption similarity is high, as could be expected (Figure 6).

## 3. Discussion

The consumption of wild plants from the Asteraceae family is generally well documented in all studied countries (Table 1).

Only dandelion is reported “consumed wild” in all 13 study sites. Several species of *Taraxacum* are used (Table 1). Interestingly, the consumption of dandelion in Greece is reported in only one publication. At the same time, *Taraxacum* is the genus with the highest number of species used in Greece, Spain and Italy [39]. Additionally, dandelion is obviously a popular wild green in Greece. There are plenty of recipes for preparing “horta” found on Internet sites which include dandelion greens (πικραλίδα or ραδίκα in Greek) together with other greens, such as amaranth, mustard greens, and chicory. This indicates that ethnobotanical studies in Greece regarding wild food plants are still insufficient.

*Cichorium intybus* L. is the second most popular species. It is reported to be used raw in salads or cooked in eleven study sites, all except Crete and Jordan and Palestine (Table 1). This plant is well known as a medicinal plant and a coffee substitute [65,66].

*Sonchus oleraceus* L. is reported as gathered and consumed in ten of the study sites, namely Albania, Bulgaria, Crete, Cyprus, Spain, southern Italy, Sicily, Jordan, Syria, and Turkey (Table 1). This plant is distributed in all 13 study sites [14]. Interestingly, for Morocco, it is reported that the related but endemic taxon *S. pinnatifidus* and not the common one is used (Table 1).

*Silybum marianum* (L.) Gaertn. is consumed raw or cooked in 9 of the 13 study sites (Table 1). This plant is distributed in all 13 study sites [14]. The Milk thistle is a popular medicinal plant [67]. Concerning the traditional use of wild food plants, most publications present qualitative data [16,17,18,19,20,21,22,23,24,25,26,27,28,29,30,31,32,33,34,35,36,37,38,39,40,41,42,43,44,45,46,47,48,49,50,51,52,53,54,55,56,57,58,59,60,61,62,63,64]. They only mark the presence or absence of consumption in certain regions and communities, but refrain from evaluating the extent of gathering and knowledge. Such evaluation is rarely performed and reveals that in some parts of our study sites, such as the Taounate Region in northern Morocco, the knowledge and use of *S. marianum* exist, but they are limited [68].

*Scolymus hispanicus* L. is reported as a plant gathered for food in eight of the study sites (Spain, Morocco, southern Italy, Albania, Greece, Crete, Cyprus, and Turkey, Table 1), but the parts used and methods of preparation are different (Table 1). The plant in Spain is so popular as food that it has recently been introduced into cultivation [69].

*Lactuca serriola* L. is consumed in seven of the study sites (Albania, Armenia, Bulgaria, Turkey, Crete, southern Italy and Spain, Table 1). Interestingly, this species is listed among ten plants prohibited in the temple in Ptolemaic Egypt due to its aphrodisiac and psychoactive properties [70]. However, there is no scientific proof for this, and on the contrary, the seeds of this plant are recommended for reducing libido [71].

*Reichardia picroides* (L.) Roth is consumed in seven countries—southern Italy, Sicily, Greece and Turkey, as well as Spain and Morocco (Table 1). Two other species of this genus are also consumed in Spain (Table 1).

*Cynara cardunculus* L. is traditionally gathered from wild populations and consumed in five countries: Spain, Morocco, southern Italy, Sicily and Cyprus (Table 1). Its native distribution also extends to Albania, Crete, and Turkey [14]. Cardoon, also known as artichoke, is a well-known cultivated vegetable with several varieties [72]. Lately, a tendency for multiuse is recommended [73], and cultivation has expanded [74].

*Centaurea calcitrapa* L. is gathered for consumption in five countries—Albania, Cyprus, Spain, Sicily, and Syria, although it is distributed in all study sites except Armenia (Table 1). If the consumption is referred to at the genus level, then it spreads to nine countries with several other species of *Centaurea* (Table 1).

*Leontodon tuberosus* L. is consumed in five countries—Greece, Crete, southern Italy, Sicily and Spain, although it is distributed in all study sites except Armenia (Table 1).

*Arctium lappa* L. is consumed in three countries that were part of the former Ottoman Empire—Armenia, Bulgaria, and Turkey. All parts of greater burdock are used raw in salads or cooked in Armenia and Bulgaria, while in Turkey, only the leaves of *A. lappa* subsp. *platylepis* (Boiss. & Balansa) are used to roll “sarma” (Table 1).

Interestingly, Armenia has a comparatively low proportion of Asteraceae consumption. The possible explanation may not be due to cultural exchange routes, but traditions in taste values. Armenians were found to dislike bitter greens [56].

## 4. Materials and Methods

The focus of this study was on wild plants from the Asteraceae family that are traditionally used as food across the territories of 11 countries (including the mainland and three islands; see Figure 1) within the Mediterranean region and adjacent areas. The selection of regions was based on principles of historical unity (e.g., the Roman Empire, Byzantine Empire, Visigothic Kingdom, Ostrogothic Kingdom, Umayyad Caliphate, Ottoman Empire, Kingdom of Aragon, Kingdom of Sicily, etc.) [75], as well as considerations of potential cultural exchange, geographic proximity, phytoclimatic similarities, and floristic relationships.

To identify relevant literature published between 1990 and 2022, we conducted searches in Google Scholar, Web of Science, and PubMed using a combination of country names (“Spain”, “France”, “Morocco”, “Sicily”, “Italy”, “Albania”, “Greece”, “Crete”, “Cyprus”, “Turkey”, “Bulgaria”, “Armenia”, “Egypt”, “Syria”, “Jordan and Palestine”, “Kosovo”, “north Macedonia”) and keywords such as “traditionally”, “wild”, “food”, “plants”, “ethnobotany”, etc. The countries were selected based on territorial proximity as well as historical and cultural interrelations.

Following the PRISMA 2000 guidelines [76], all records were screened for eligibility. A total of 561 publications were excluded for the following reasons: the information was not relevant to the research topic; the data pertained solely to medicinal uses of plants; the studies addressed traditional food practices but did not include wild plants; the records referred exclusively to the consumption of cultivated plants.

### 4.1. Distribution of Traditionally Consumed Wild Plants from the Asteraceae Family 

From the selected publications, we extracted information on wild plants from the Asteraceae family that are traditionally used as food in the following countries and regions: Spain [23,24,25], Morocco [16,17,18,19,20,21,22], Sicily [26,27,28,29], southern Italy [30,31,32,33,34], Albania [32,35,36], Greece [37,38], Crete [37,39,40], Cyprus [37,40], Turkey (Türkiye) [41,42,43,44,45,46], Bulgaria [42,47,48,49,50,51], Armenia [52,53,54,55,56,57,58,59], Syria [60], and Jordan and Palestine [61,62,63,64]. We detected a potential bias in the source data, such as overrepresentation of some regions due to more intensive fieldwork or publication rates. For example, the studies in Spain, Morocco, southern Italy, and Turkey exceed the research performed in Syria and Jordan and Palestine. Also, there is an indication that ethnobotanical studies in Greece regarding wild food plants are still insufficient, considering the irrelevant presentation of dandelion consumption. It was found that, for France, Egypt, Kosovo, and northern Macedonia, there is a lack of studies and publications on the traditional use of wild food plants, and in particular, from the Asteraceae family, rendering the available data inadequate for inclusion in this investigation. The presentation of the consumption methods is rather heterogeneous in the published sources [16,17,18,19,20,21,22,23,24,25,26,27,28,29,30,31,32,33,34,35,36,37,38,39,40,41,42,43,44,45,46,47,48,49,50,51,52,53,54,55,56,57,58,59,60,61,62,63,64]. Therefore, this information is summarized in Table 1, but is not used for further analysis and interpretation.

### 4.2. Data Set Preparation and Analyses

We organized the reported data for each country in Excel tables. The distribution range of each plant taxon in the territories of the studied areas was added to the tables following Euro+Med Plantbase [14]. Additionally, the distribution range was double-checked following the Plants of the World Online [77] as we noticed that some species were reported in error in the Euro+Med Plantbase for some of the areas included in this study. Also, some mistakes in the published data were noticed and eliminated in the tables and analyses. For instance, *Helianthus tuberosus* L., reported to be gathered and consumed in Turkey [43], is actually an introduced species in this country, naturalized in Albania, Bulgaria, Cyprus, southern Italy, Sicily, Spain, and cultivated in Greece and Palestine [14]. Also, *Arctium tomentosum* Mill., which is documented as a food plant only in Armenia [52], is native in all studied countries except Armenia and Spain, where it is an introduced species [14]. They are not included in Table 1, nor in the analysis.

Particular taxa are casual (alien species that do not form self-sustaining populations in the invaded region) or naturalized in some countries, and these cases are removed from the data set.

We summarized the data about the plant parts utilized and the modes of consumption, as reported in the original sources (Table 1). Due to inconsistencies in the precision and detail of these descriptions, standardization of plant part usage was not feasible. Consequently, the resulting data matrix did not support in-depth analyses of convergent or divergent plant uses, nor allow for robust interpretations of cultural exchange. Where original publications included synonyms, these were replaced with the currently accepted taxonomic names following [14], which were subsequently used for basic descriptive statistical analysis

#### Jaccard Similarity Coefficient or Jaccard Index JI

Jaccard similarity coefficient or JI is used when the level of similarity between two groups of elements needs to be identified [78]. We used JI to evaluate the similarity of use and the similarity of distribution among all possible country pairs. JI is calculated using the following formula:

JI [%] = N_AB_ ∗ 100/(N_A_ + N_B_ − N_AB_)

where

N_A_ is the number of elements in study site A (country/mainland or island)
N_B_ is the number of elements in study site B (country/mainland or island)
N_AB_ is the number of elements available in both study sites (country/mainland or island)

## 5. Conclusions

This work focused on the wild plants of the Asteraceae family, which are traditionally used as food in some Mediterranean countries. It has provided an overview of the most commonly consumed members of this family, highlighting similarities and differences between the territories of reference. In particular, this survey revealed 167 specific and infraspecific taxa from the Asteraceae family used traditionally as food in 11 Mediterranean countries and three islands. This traditional knowledge about edible wild plants is a priceless heritage that should be passed to future generations. The working statement that neighboring countries have high similarity in the distribution pattern of edible wild Asteraceae species is confirmed by the Jaccard index statistical model. However, the consumption of these plants does not follow the distribution pattern. This indicates the important role of the cultural exchange, but tracing it is too complicated and practically impossible. Our results are a basis for future pharmacological and phytochemical studies that will contribute to sustainable food systems in the Mediterranean. It points to wild plant candidates for domestication or agroecological innovations in food production. These are *Scolymus hispanicus*, *Cynara cardunculus*, *Cichorium intybus*, as well as other traditionally popular plants.

## Figures and Tables

**Table 1:** Wild plants from the Asteraceae family traditionally used as food, and their distribution in 11 Mediterranean countries and adjacent territories. Legend: JO—Jordan and Palestine; IT—southern Italy; MA—Morocco; Sicily; ES—Spain; SY—Syria; TR—Turkey; AL—Albania; AM—Armenia; BG—Bulgaria; Crete; CY—Cyprus; GR—Greece. Note: asterisk marks those species that are collected in the field and eaten only in one or two of the 50 Spanish provinces, while all other species listed for Spain are used in three or more provinces. The distribution of the taxa basically follows the Euro+Med Plantbase [14]; double asterisks mark the synonym names used in the original publications.

Taxon	Presence in the Study Sites	Used in the Study Sites	Used Parts and Modes of Application as Food
*Achillea millefolium* L.	AL, AM, BG, ES, GR, IT, SY, TR	AM IT	Leaves, stems, flowers Leaves, soups, omelets
2. *Achillea nobilis* L.	AL, AM, BG, ES, GR, IT, TR	TR	Aboveground, hot drink
3. *Achillea tenuifolia* Lam.	AM, TR	AM	Leaves, stems, flowers
4. *Anacyclus clavatus* (Desf.) Pers.	AL, ES, GR, IT, MA, Sicily, TR	ES	Tender leaves and stems, raw in salads or stewed
5. *Anacyclus valentinus* L.	ES, MA	* ES	Tender stems and leaves, stewed
6. *Andryala integrifolia* L.	Crete, ES, GR, IT, MA, Sicily, TR	ES	Tender stems, raw as a snack
7. *Andryala laxiflora* DC.	ES, MA	* ES	Tender stems, raw
8. *Anvillea radiata* Coss. & Durieu	MA	MA	Aerial parts
9. *Arctium lappa* L.	AL, AM, BG, Crete, CY, ES, GR, IT, TR	AM BG	Stems, leaves, roots Root, leaves, shoots, salad raw, soup, coffee substitute/beverage
10. *Arctium lappa* subsp. *platylepis* (Boiss. & Balansa) Arènes	TR	TR	Leaves, “sarma”
11. *Arctium minus* (Hill) Berhn.	AL, BG, CY, ES, GR, IT, JO, MA, Sicily, SY, TR	ES TR	Basal leaves and stems, stewed Leaves, “sarma”
12. *Artemisia absinthium* L.	AL, AM, BG, Crete, ES, GR, IT, MA, TR	AM TR	Leaves Leaves, roasted
13. *Artemisia annua* L.	AL, AM, BG, GR, SY, TR	AM	Leaves
14. *Artemisia atlantica* Coss. & Durieu	MA	MA	Aerial parts
15. *Artemisia campestris* L.	AL, BG, ES, GR, IT, MA, Sicily, TR	* ES	Aerial parts, for preserving raisins
16. *Artemisia herba-alba* Asso.	ES, MA	MA	Aerial parts
17. *Artemisia vulgaris* L.	AL, AM, BG, ES, GR, Sicily, TR	BG	Leaves, pastries,
18. *Atractylis cancellata* L.	Crete, CY, ES, GR, IT, JO, MA, Sicily, SY, TR	Crete	Flower receptacles, raw or boiled
19. *Bellis perennis* L.	AL, AM, BG, Crete, CY, ES, GR, IT, JO, MA, Sicily, SY, TR	BG Sicily	Leaves, salad raw, soup Tender leaves of the basal rosette, raw in salads or simply stewed, or as an ingredient in soups
20. *Brocchia cinerea* (Delile) Vis.	JO, MA,	MA	Aerial parts before flowering
21. *Calendula arvensis* (Vaill.) L.	AL, AM, BG, Crete, CY, ES, GR, IT, JO, MA, Sicily, SY, TR	CY, * ES GR MA	Leaves, boiled Basal leaves, stewed Leaves, boiled Aerial parts
22. *Carduncellus dianius* Webb (Syn. ** *Carthamus dianius* (Webb) Coincy)	ES	* ES	Basal leaves, raw or stewed
23. *Carduus hamulosus* Ehrh.	AL, AM, BG, GR, TR	AM	Stems, leaves
24. *Carduus meonanthus* Hoffmanns. & Link	ES, MA	* ES	Basal leaves, stewed
25. *Carduus nutans* L.	AL, AM, BG, ES, GR, IT, MA, Sicily, SY, TR	BG GR IT	Sprouts, young herbage spines removed, stew Aerial part, soups, boiled, and then stir-fried Aerial part, boiled
26. *Carduus tenuiflorus* Curtis	ES, IT, MA, Sicily	* ES	Basal leaves and tender stems, stewed
27. *Carlina acanthifolia* All.	AL, BG, ES, GR, IT	* ES	Immature inflorescences, raw as a snack
28. *Carlina acaulis* L.	AL, BG, Crete, ES, GR, IT	IT	Young leaves, capitula (inflorescences), boiled in mixtures, fried
29. *Carlina corymbosa* L.	AL, BG, Crete, ES, GR, IT, TR	IT	Young leaves, boiled in mixtures
30. *Carlina gummifera* (L.) Less. (Syn. ** *Atractylis gummifera* L.)	Crete, ES, GR, IT, MA, Sicily, TR	* ES MA Sicily	Tender sprouts with young leaves, stewed Roots Fleshy receptacle of the capitula (inflorescences), raw or stewed
31. *Carlina sicula* Ten.	IT, Sicily	Sicily	Tender stems, stewed, seasoned with oil and lemon, or fried with eggs
32. *Carlina vulgaris* L.	AL, AM, BG, ES, GR, IT, TR	IT	Young leaves boiled in mixtures
33. *Carthamus lanatus* L.	AL, AM, BG, Crete, CY, ES, GR, IT, JO, MA, Sicily, TR	ES	Basal leaves, raw in salads or stewed
34. *Centaurea aspera* L.	ES, IT, MA, Sicily	* ES	Inflorescences, stewed
35. *Centaurea calcitrapa* L.	AL, BG, Crete, CY, ES, GR, IT, JO, MA, Sicily, SY, TR	AL CY * ES Sicily SY	Young leaves, boiled in mixtures Young rosettes, cooked, especially with beans Basal leaves and tender stems, stewed Tender leaves of the basal rosette, boiled and simply seasoned with salt and olive oil Young aerial part, steamed with “seleeg”
36. *Centaurea cyanus* L.	AL, AM, BG, Crete, ES, GR, IT, Sicily, TR	BG	Young herbage
37. *Centaurea diluta* Aiton	ES, MA	MA	Stems
38. *Centaurea dumulosa* Boiss.	JO, SY	JO	Stem, eaten cooked or raw as salads
39. *Centaurea iberica* Spreng.	AM, BG, CY, GR, IT, JO, Sicily, SY, TR	JO	Stems
40. *Centaurea napifolia* L.	IT, MA, Sicily	Sicily	Boiled basal leaves, alone or with other wild greens, are eaten with olive oil, salt, and lemon juice
41. *Centaurea sicula* L.	ES, IT, MA, Sicily	IT	Young leaves, boiled in mixtures
42. *Centaurea solstitialis* L.	AL, AM, BG, Crete, CY, ES, GR, IT, JO, MA, Sicily, SY, TR	IT	Young leaves, boiled in mixtures, eaten with olive oil, salt, and lemon juice
43. *Chondrilla juncea* L.	AL, AM, BG, Crete CY, ES, GR, IT, JO, MA, Sicily, SY, TR	AL ES IT	Young shoots raw, as a snack with bread Young shoots and basal leaves, raw in salads or stewed Young shoots raw, as a snack with bread
44. *Chrysanthoglossum trifurcatum* (Desf.) B. H. Wilcox & et al.	MA	MA	Aerial parts
45. *Cichorium intybus* L.	AL, AM, BG, Crete, CY, ES, GR, IT, JO, MA, Sicily, SY, TR	AL AM BG CY ES GR IT MA Sicily SY TR	Basal rosettes, shoots blanched and fried on pasta, or with eggs; in boiled mixtures Leaves, seeds Roots, leaves, shoots, salad raw, soup, coffee surrogate/beverage Rosettes, in mixed salads or boiled Roots toasted, as a coffee substitute Rosettes, in mixed salads or boiled Basal rosettes, shoots blanched and fried on pasta, or with eggs; in boiled mixtures Leaves Basal rosette leaves, raw in salads or stewed Young aerial part, “shabshuleh”: boiled, then olive oil and lemon juice, and garlic are added; steamed with “seleeg” Aboveground parts meal, salad
46. *Cichorium pumilum* Jacq.	AL, BG, Crete, CY, ES, GR, IT, JO, MA, Sicily, SY, TR	CY JO	Leaves, boiled Leaves, boiled and eaten as salad with yoghurt
47. *Cichorium spinosum* L.	Crete, CY, ES, GR, IT, Sicily, TR	Crete	Whorls, in mixed salads or boiled in mixtures
48. *Cirsium arvense* (L.) Scop.	AL, AM, BG, ES, GR, IT, JO, Sicily, TR	* ES TR	Young shoots, stewed Leaves, meal, stuffed
49. *Cirsium pubigerum* (Desf.) DC.	AM, TR	TR	Eaten fresh
50. *Cirsium vulgare* (Savi) Ten.	AL, AM, BG, Crete, CY, ES, GR, IT, JO, MA, Sicily, SY, TR	SY	Leaves, midrib and underground stem
51. *Cota palaestina* Kotschy (Syn. ** *Anthemis palaestina *(Kotsky) Boiss.).	Crete, CY, JO, SY, TR	JO	Inflorescence, herbal tea
52. *Cota tinctoria* (L.) J. Gay (Syn. ** *Anthemis tinctoria* L.)	AL, AM, BG, CY, GR, IT, JO, Sicily, SY, TR	TR	Inflorescence, herbal tea
53. *Crepis apula* (Fiori) Babc.	IT	IT	Aerial parts, boiled
54. *Crepis commutata* (Spreng.) Greuter	BG, Crete, CY, GR, SY, TR	Crete	Rosettes, boiled in mixtures
55. *Crepis leontodontoides* All.	AL, IT, Sicily	Sicily	Basal rosette leaves, boiled and simply seasoned with salt and olive oil
56. *Crepis sancta* (L.) Bornm.	AL, AM, BG, Crete, CY, ES, GR, IT, JO, Sicily, SY, TR	BG IT SY	Young herbage Aerial part, boiled Young aerial part, steamed with “seleeg”; steamed with chickpea
57. *Crepis setosa* Haller f.	AL, BG, ES, GR, IT, Sicily, TR	IT	Aerial part, boiled
58. *Crepis vesicaria* L. subp. *taraxacifolia* (Thuill.) Thell.	AL, ES, GR, IT, MA, Sicily	AL IT	Young leaves, boiled in mixtures Young leaves, boiled in mixtures, fried, raw: mixed salads
59. *Crepis vesicaria* L. subsp. *vesicaria*	AL, Crete, ES, GR, IT, MA, Sicily, TR	Sicily ES	Basal rosette leaves, raw in salads or simply stewed, or as an ingredient in soups and omelets Basal leaves, raw in salads or stewed
60. *Crepis vesicaria* L.	AL, Crete, ES, GR, IT, MA, Sicily, SY, TR	Crete IT SY	Rosettes, boiled in mixtures Aerial part, boiled Young aerial part, steamed with “seleeg”
61. *Cynara cardunculus* L.	AL, Crete, CY, ES, GR, IT, MA, Sicily, TR	CY ES IT MA Sicily	Flower receptacles and stems, raw (consumed with lemon and salt), pickled, cooked Inflorescences and basal leaves, stewed Stems, basal rosettes, boiled and then deep-fried Inflorescences, roots, dried or peeled Tender leaves and parts of the stem, stewed or fried in batter; capitula (inflorescences) for preserves in olive oil or vinegar
62. *Cynara cornigera* Lindl.	Crete, CY, GR	Crete CY GR	Young stems and flower receptacles, flower receptacles: raw in salads; young stems: cooked, often together with lamb or goat meat Flower receptacles and stems, raw (consumed with lemon and salt), pickled, cooked Flower receptacles and stems, raw (consumed with lemon and salt), pickled, cooked
63. *Cynara humilis* L.	ES, MA	ES MA	Inflorescences, stewed Inflorescence, steamed with minced meat
64. *Cynara syriaca* Boiss.	JO, SY	SY	Inflorescence, steamed with minced meat
65. *Cynara tournefortii* Boiss. & Reut.	ES, MA	ES	Inflorescences, stewed
66. *Dittrichia graveolens* (L.) Greuter	AL, AM, BG, Crete, CY, ES, GR, IT, JO, MA, Sicily, SY, TR	* ES	Aerial parts, for preserving raisins
67. *Dittrichia viscosa* (L.) Greuter	AL, AM, BG, Crete, CY, ES, GR, IT, JO, MA, Sicily, SY, TR	* ES	Aerial parts, for preserving raisins and potatoes
68. *Doronicum orientale* Hoffm.	AL, BG, Crete, GR, IT, Sicily, SY, TR	BG	Leaves
69. *Echinops heterophyllus* P.H. Davis	TR	TR	Eaten fresh; fresh plant is eaten after peeling off the outer part
70. *Echinops orientalis* Trautv.	AM, TR	TR	Eaten fresh
71. *Echinops spinosissimus* Turra	AL, Crete, CY, GR, IT, JO, MA, Sicily, SY, TR	CY GR MA	Young stems, raw as snacks or in salads Young stems, raw as snacks or in salads Roots peeled as well as roots dried, receptacles (seldom)
72. *Endopappus macrocarpus* Sch. Bip.	MA	MA	Leaves
73. *Gelasia hirsuta* (Gouan) Zaika, Sulhor. (syn. ** *Lasiospora hirsuta* (Gouan) Cass.; *Scorzonera hirsuta* (Gouan) L.)	ES, IT, Sicily	IT	Raw as a snack, with bread, or in mixed vegetables
74. *Gelasia villosa* (Scop.) Coss. (Syn. ** *Scorzonera villosa* Scop.)	AL, IT, Sicily	IT	Basal rosettes, young stems, raw as a snack, or in boiled mixtures
75. *Geropogon hybridus* (L.) Sch. Bip. (Syn. ** *Tragopogon hybridus* L.)	AL, Crete, CY, ES, GR, IT, JO, MA, Sicily, SY, TR	* ES JO MA	Tender leaves and stems, raw as a snack Leaves Leaves
76. *Glebionis coronaria* (L.) Spach (Syn. ** *Chrysanthemum coronarium* L.)	Crete, CY, ES, GR, IT, JO, MA, Sicily, SY, TR	Crete * ES JO MA	Young aerial parts, raw in mixed salads or in boiled mixtures Tender leaves and stems, stewed Inflorescences, herbal tea Stems
77. *Glebionis segetum* (L.) Fourr. (Syn. ** *Chrysanthemum segetum* L.)	AL, Crete, CY, ES, GR, IT, JO, MA, Sicily, SY, TR	Crete CY IT	Young aerial parts, raw in mixed salads or in boiled mixtures Young shoots, pickled Top of aerial part, raw as snacks
78. *Gundelia tournefortii* L.	AM, CY, JO, SY, TR	JO SY TR	Young stems, inflorescence, cooked with meat and yoghurt Leaves midrib and underground stem Leaves, roasted, salad, pickle, consumed as coffee; cooked plant with yogurt; obtained gum chewed; used in cheese production
79. *Hedypnois rhagadioloides* (L.) F. W. Schmidt	AL, AM, BG, Crete, CY, ES, GR, IT, JO, MA, Sicily, SY, TR	* ES IT	Basal leaves, raw in salads Basal leaves, cooked
80. *Helichrysum italicum* (Roth) G. Don	Crete, CY, ES, GR, IT, MA, Sicily, TR	ES	Inflorescences in olive oil, for seasoning roasted meat
81. *Helichrysum rubicundum* (K. Koch) Bornm.	AM, TR	AM	Flowers, leaves
82. *Helminthotheca comosa* (Boiss.) Holub (Syn. ** *Picris comosa* (Boiss.) B. D. Jacks.	ES, MA	ES	Basal leaves, stewed
83. *Helminthotheca echioides* (L.) Holub (Syn. ** *Picris echioides* L.)	AL, BG, Crete, CY, GR, ES, IT, JO, MA, Sicily, SY, TR	AL Crete GR * ES IT Sicily SY	Aerial parts, boiled Young rosettes, in mixtures, boiled Aerial parts, boiled Basal leaves, stewed Basal rosettes, raw or boiled in mixtures, salads, soups Basal leaves, stewed with other vegetables, then sautéed and seasoned with garlic, olive oil, chili and lemon juice Young aerial parts, steamed with chickpea and olive oil; steamed with onion and olive oil; steamed with minced meat; cooked with rice
84. *Hirschfeldia incana* (L.) Lagr.-Foss.	AL, AM, Crete, CY, GR, ES, IT, JO, MA, Sicily, SY, TR	Crete	Young aerial parts, in mixtures, boiled or in pies
85. *Hyoseris radiata* L.	Crete, ES, IT, MA, Sicily, TR	IT Sicily	Inflorescences raw as a snack, with bread, or in salads Basal leaves, boiled and simply seasoned with salt and olive oil, or as an ingredient in soups
86. *Hypochaeris achyrophorus* L.	AL, Crete, CY, GR, ES, IT, JO, MA, Sicily, SY, TR	IT Sicily	Young leaves, boiled in mixtures Basal rosette leaves, boiled and simply seasoned with salt and olive oil
87. *Hypochaeris cretensis* (L.) Bory & Chaub.	AL, BG, Crete, GR, IT, Sicily	Sicily	Basal rosette leaves, boiled and simply seasoned with salt and olive oil
88. *Hypochaeris laevigata* (L.) Ces. & et al.	ES, IT, MA, Sicily	IT	Young leaves, boiled in mixtures
89. *Hypochaeris radicata* L.	AL, BG, Crete, GR, ES, IT, MA, Sicily, TR	ES IT Sicily	Basal leaves, raw or stewed Young leaves, boiled in mixtures Basal rosette leaves, raw in salads; blanched or stewed as an ingredient in soups; omelets
90. *Inula helenium* L.	AL, AM, BG, GR, IT, TR	AM BG	Leaves, flowers Roots, soup, beverages
91. *Lactuca saligna* L.	AL, BG, Crete, CY, GR, ES, IT, JO, MA, Sicily, SY, TR	ES JO	Young shoots and basal leaves, raw in salads Leaves, eaten raw as salad, or eaten raw
92. *Lactuca serriola* L.	AL, AM, BG, Crete, CY, ES, GR, IT, JO, MA, Sicily, SY, TR	AL AM BG Crete * ES IT TR	Aerial parts, boiled in mixtures Stems Sprouts and young leaves, salad raw, soup Young leaves, in mixtures, boiled Young shoots and basal leaves, raw in salads Basal rosettes, boiled in mixtures Leaves, salads
93. *Lactuca viminea* (L.) J. Presl & C. Presl	AL, AM, BG, Crete, CY, ES, GR, IT, JO, MA, Sicily, SY, TR	ES IT	Young shoots and basal leaves, raw in salads
94. *Lapsana communis* L.	AL, AM, BG, ES, GR, IT, JO, MA, Sicily, SY, TR	BG IT	Leaves Basal leaves, raw as salad
95. *Launaea capitata* (Spreng.) Dandy	JO, MA	MA	Aerial parts
96. *Launaea fragilis* (Asso) Pau	CY, ES, JO, MA, Sicily	MA	Aerial parts
97. *Launaea lanifera* Pau	ES, MA	MA	Leaves
98. *Launaea nudicaulis* (L.) Hook f.	ES, JO, MA, Sicily, SY	* ES JO MA	Young shoots and basal leaves, raw in salads or stewed Above-ground organs Aerial parts
99. *Leontodon saxatilis* Lam.	AL, BG, ES, IT, MA, TR	ES MA	Basal leaves, raw in salads or stewed Leaves
100. *Leontodon tuberosus* L.	AL, BG, Crete, CY, ES, GR, IT, JO, MA, Sicily, SY, TR	Crete * ES GR IT Sicily	Young leaves and roots Basal leaves, raw in salads or stewed; tubers, raw as a snack Aerial parts, boiled Aerial parts, boiled Basal rosette leaves, boiled and simply seasoned with salt and olive oil
101. *Leucanthemum vulgare* Lam.	AM, ES, GR, IT, TR	AM	Leaves, flowers
102. *Limbarda crithmoides* (L.) Dumort. (Syn. ** *Inula crithmoides* L.)	AL, Crete, CY, ES, GR, IT, JO, MA, Sicily, SY, TR	JO	Leaves
103. *Mantisalca salmantica* (L.) Briq. & Cavill.	CY, ES, GR, IT, JO MA, Sicily, TR	ES	Basal leaves, raw or stewed
104. *Matricaria aurea* (Loefl.) Sch. Bip.	Crete, CY, ES, JO, MA, Sicily, SY, TR	JO	Inflorescence, herbal tea
105. *Matricaria chamomilla* L.	AL, BG, Crete, CY, ES, GR, IT JO, MA, Sicily, SY, TR	BG GR SY TR	Inflorescence, herbal tea Inflorescence, herbal tea Inflorescence, herbal tea Inflorescence, herbal tea
106. *Notobasis syriaca* (L.) Cass.	AL, AM, Crete, CY, ES, GR, IT, JO, MA, Sicily, SY, TR	CY GR JO SY	Aerial parts, raw and boiled Aerial parts, boiled Stems, eaten raw Leaves midrib, steamed with onion and olive oil; boiled, then steamed with minced meat
107. *Onopordum acanthium* L.	AL, AM, BG, ES, GR, IT, TR	AM BG * ES	Roots, stems, flowers Roots, leaves, shoots (less than 20 cm tall), salad raw, soup, coffee surrogate/beverage Tender basal leaves, stewed
108. *Onopordum acaulon* L.	ES, MA	* ES	Tender basal leaves, stewed
109. *Onopordum carduiforme* Boiss.	JO, SY	JO	Stems, eaten raw
110. *Onopordum corymbosum* Willk.	ES	* ES	Tender basal leaves, stewed
111. *Onopordum cyprium* Eig	CY	CY	Young stems, raw as snacks or in salads
112. *Onopordum illyricum* L.	AL, BG, Crete, ES, GR, IT, JO, Sicily, SY, TR	IT Sicily	Basal rosettes, shoots soups; roots, boiled then fried Leaf veins and the basal part of the tuft of leaves and roots, stewed or fried in batter
113. *Onopordum macracanthum* Schousb.	ES, MA	* ES	Tender basal leaves, stewed
114. *Onopordum nervosum* Boiss.	ES	ES	Tender stems and basal leaves, stewed
115. *Onopordum tauricum* Willd.	AL, BG, Crete, GR, IT, SY, TR	TR	Stem salad
116. *Otoglyphis pubescens* (Desf.) Pomel (Syn. ** *Aaransolina pubescens* (Desf. & Humphries)		MA	Leaves
117. *Petasites hybridus* (L.) G. Gaertn. & et al.	AL, AM, BG, ES, GR, IT, Sicily, TR	BG TR	Leaves, salad, soup, stew Leaves, “sarma”
118. *Phagnalon saxatile* (L.) Cass.	AL, Crete, ES, GR, IT, MA, Sicily	Crete	Young leaves, in mixtures, boiled
119. *Picris hieracioides* L.	AL, AM, BG, ES, GR, IT, Sicily, SY, TR	IT	Basal rosettes, boiled in mixtures
120. *Pseudopodospermum crispatulum* (DC.) Zaika, Sukhor. & N. Kilian (Syn. ** *Scorzonera crispatula* (DC.) Boiss.)	ES	ES	Basal leaves, raw in salads
121. *Pseudopodospermum hispanicum* (L.) Zaika, Sukhor. & N. Kilian (Syn. ** *Scorzonera hispanica* L.)	AL, BG, ES, GR, IT, MA, TR	* ES	Tender stems and basal leaves, raw in salads
122. *Pseudopodospermum molle* (M. Bieb.) Kuth. (Syn. ** *Scorzonera mollis* M. Bieb.)	AL, BG, Crete, GR, TR	TR	Eaten fresh; leaves cooked as a vegetable
123. *Pseudopodospermum papposum* (DC.) Zaika, Sukhor. & N. Kilian (Syn. ** *Scorzonera papposa* DC.)	JO, SY, TR	JO	Stem and leaves, raw as salad, eaten raw
124. *Pseudopodospermum undulatum* (Vahl) Zaika, Sukhor & N. Kilian (Syn. ** *Scorzonera undulata* Vahl)	MA, Sicily	MA	Leaves, roots, heads
125. *Raponticum acaule* (L.) DC.	MA	MA	Bottom of receptacles
126. *Reichardia intermedia* (Sch. Bip.) Samp.	Crete, ES, CY, GR, JO, MA, Sicily, TR	* ES	Basal leaves, raw in salads or stewed
127. *Reichardia picroides* (L.) Roth	AL, BG, Crete, CY, ES, GR, IT, JO, MA, Sicily, SY, TR	AL Crete ES GR IT MA Sicily	Basal rosettes, raw in salads, or boiled Aerial parts, in mixed salads, or in mixtures, boiled Basal leaves, raw in salads or stewed Leaves, boiled Basal rosettes, raw in salads or boiled Aerial parts Basal rosette leaves, raw in salads or stewed, or as an ingredient in soups
128. *Reichardia tingitana* (L.) Roth	Crete, CY, ES, GR, IT, JO, MA, Sicily, SY, TR	* ES	Basal leaves, raw in salads or stewed
129. *Rhagadiolus stellatus* (L.) Gaertn.	AL, BG, Crete, CY, ES, GR, IT, JO, MA, Sicily, SY, TR	* ES IT	Basal leaves, raw in salads or stewed Basal rosettes, boiled in mixtures; boiled and then fried
130. *Scolymus grandiflorus* Desf.	IT, MA, Sicily, TR	Sicily	Tufts of basal leaves, stewed or fried in batter
131. *Scolymus hispanicus* L.	AL, BG, Crete, CY, ES, GR, IT, JO, MA, Sicily, SY, TR	AL Crete CY ES GR IT MA TR	Leaf stalks, cooked with eggs and cheese Young shoots, tender peduncles and rachis of leaves (sometimes with parts of the stems), underground part of the stems, and external coats of the roots, cooked alone with eggs and goat or lamb meat Young shoots, tender peduncles, and rachis of leaves (sometimes with parts of the stem); the underground part of stems and external coat of the roots, cooked Leaf stalks, cooked with eggs and cheese Tender stems and basal leaves, roots peeled as well as dried Root stew, meal
132. *Scolymus maculatus* L.	BG, Crete, CY, JO, ES, GR, IT, MA Sicily, SY, TR	* ES JO MA	Basal leaves and young stems (leaf rosettes), raw in salads or stewed Leaves and stems Stems after taking away spines
133. *Scorzonera angustifolia* L.	ES, MA	ES	Tender stems and basal leaves, raw in salads
134. *Scorzonera laciniata* L. (Syn. ** *Podospermum laciniatum* (L.) DC.)	AL, AM, BG, CY, ES, GR, IT, MA, Sicily, TR	AM ES	Roots, leaves Tender stems and basal leaves, raw in salads
135. *Scorzonera schweinfurthii* Boiss.	JO	JO	Leaves
136. *Scorzoneroides cichoriacea* (Ten.) Greuter	AL, BG, GR, IT, Sicily, TR	Sicily	Basal rosette leaves, boiled and seasoned with salt and olive oil
137. *Senecio flavus* (Decne.) Sch. Bip.	ES, JO, MA, SY	JO	Roots
138. *Senecio leucanthemifolius* subsp. vernalis (Waldst. & Kit.) Greuter	AL, AM, BG, Crete, CY, ES, GR, IT, JO, MA, Sicily, SY, TR	AM	Leaves, stems, roots
139. *Senecio vulgaris* L.	AL, AM, BG, Crete, CY, ES, GR, IT, JO, MA, Sicily, SY, TR	TR	Aboveground parts, stew
140. *Silybum marianum* (L.) Gaertn.	AL, AM, BG, Crete, CY, ES, GR, IT, JO, MA, Sicily, SY, TR	BG ES GR IT JO MA Sicily SY TR	Leaves, sprouts, young anthodia, salad, soup, stewed Seeds, raw and tender basal leaves, raw in salads or stewed Leaves, sprouts, young anthodia, salad, soup, stewed Leaves, sprouts, young anthodia, salad, soup, stewed Stem and leaves, cooked as soup, raw as salad, eaten raw Leaves Tender shoots raw in salads or as an ingredient in soups; capitula (inflorescences), receptacles, stewed Leaves midrib and underground stem, steamed with onion and olive oil; steamed with “seleeg”; steamed with chickpea and olive oil, steamed with minced meat, and then mixed with yogurt Leaves, “sarma”
141. *Sonchus arvensis* L.	AL, AM, BG, CY, ES, GR, IT, TR	BG	Leaves, salad raw, soup
142. *Sonchus asper* (L.) Hill	AL, AM, BG, Crete, CY, ES, GR, IT, JO, MA, Sicily, SY, TR	AL BG ES IT MA Sicily	Leaves, salad raw, soup Leaves, salad raw, soup Basal leaves and tender stems, raw in salads or stewed Young aerial parts, boiled, alone or in mixtures Leaves, stems Basal leaves, raw in salads or boiled and simply seasoned with salt and olive oil
143. *Sonchus bulbosus* (L.) N. Kilian & Greuter (Syn. ** *Aetheorhiza bulbosa* (L.) Cass.)	AL, Crete, CY, ES, GR, IT, JO, MA, Sicily, SY, TR	* ES	Bulbs, raw as a snack
144. *Sonchus crassifolius* Willd.	ES	ES	Young shoots and leaves, raw in salads
145. *Sonchus maritimus* L.	AL, AM, ES, IT, JO, MA, Sicily, SY	IT	Basal rosettes, in fish-based soups
146. *Sonchus oleraceus* L.	AL, AM, BG, Crete, CY, ES, GR, IT, JO, MA, Sicily, SY, TR	AL BG Crete CY ES IT JO Sicily SY TR	Leaves, salad raw, soup Leaves, salad raw, soup Young aerial parts, in boiled mixtures Young aerial parts, in mixed salads or boiled Young shoots and leaves, raw in salads Young aerial parts, boiled, alone or in mixtures Shoots Whole young plant or tender shoots of the adult stem raw in salads or boiled and simply seasoned with salt and olive oil Young aerial part, steamed with “seleeg” Leaves, roasted, meal, pie, salad
147. *Sonchus pinnatifidus* Cav.	MA	MA	Leaves, stems
148. *Sonchus tenerrimus* L.	Crete, CY, ES, GR, IT, JO, MA, Sicily, SY, TR	ES MA	Young shoots and leaves, raw in salads Leaves, stems
149. *Taraxacum cyprium* H. Lindb.	CY, JO	CY	Young aerial parts, in mixed salads or boiled
150. *Taraxacum getulum* Pomel	MA	MA	Leaves
151. *Taraxacum hellenicum* Dahlst.	Crete, CY, GR, SY, TR	Crete CY	Young rosettes, boiled in mixtures Young aerial parts, in mixed salads or boiled
152. *Taraxacum obovatum* (Willd.) DC.	ES, IT, MA, Sicily	ES IT MA	Basal leaves, raw in salads Basal rosettes boiled in mixtures Leaves
153. *Taraxacum officinale* aggr.	AL, AM, BG, ES, GR, IT, MA, Sicily, SY, TR	AL AM BG ES GR IT TR	Leaves (fresh), filling for börek (korimak) Leaves Leaves, young anthodia, salad, marinated anthodia Basal leaves (leaf rosette), raw in salads Basal leaves Basal rosettes, boiled in mixtures Leaves, salad, raw, stew, pancake, meal, cold drink (flower)
154. *Taraxacum sonchoides* (D. Don) Sch. Bip.	AM, SY, TR	AM	Leaves
155. *Tolpis barbata* (L.) Gaertn.	ES, MA	* ES	Basal leaves (leaf rosette), raw in salads
156. *Tragopogon buphthalmoides* (DC.) Boiss.	AM, JO, SY, TR	JO TR	Leaves Leaves, eaten fresh in salads
157. *Tragopogon crocifolius* L.	ES, IT, MA, Sicily	IT	Leaves, raw in salads
158. *Tragopogon dubius* Scop.	AL, AM, BG, Crete, ES, GR, IT, MA, TR	BG	Leaves, salad raw, soup, stew
159. *Tragopogon graminifolius* DC.	AM, TR	AM	Roots, leaves
160. *Tragopogon porrifolius* L.	AL, AM, BG, Crete, CY, ES, GR, IT, JO, MA, Sicily, SY, TR	ES IT MA TR	Tender leaves and stems, raw in salads Leaves, young stems, raw as a snack, or in mixed salads Roots, leaves Shoots and leaves, salads, stewed with yogurt
161. *Tragopogon porrifolius* subsp. *longirostris* (Sch. Bip.) Greuter	AM, Crete, CY, GR, JO, SY, TR	JO	Stem, eaten raw
162. *Tragopogon pratensis* L.	AL, BG, ES, GR, IT, SY, TR	* ES SY	Tender stems, raw as a snack Young aerial part, steamed with ”seleeg”
163. *Tragopogon reticulatus* Boiss. & A. Huet	AM, TR	AM	Roots, leaves
164. *Tripolium pannonicum* (Jacq.) Dobrocz.	AL, BG, Crete, ES, GR, IT, JO, Sicily, TR	IT	Basal rosettes, shoots, in lake fish-based soups
165. *Tussilago farfara* L.	AL, AM, BG, Crete, CY, ES, GR, IT, MA, Sicily, SY, TR	BG TR	Young leaves, sprouts anthodia, salad blanched, soup, pastry Leaves, stuffed
166. *Urospermum dalechampii* (L.) F. W. Schmidt	ES, IT, MA, Sicily,	IT Sicily	Basal rosettes, boiled in mixtures Basal rosette leaves, boiled and simply seasoned with salt and olive oil
167. *Urospermum picroides* (L.) F. W. Schmidt	AL, AM, BG, Crete, CY, ES, GR, IT, JO, MA, Sicily, SY, TR	* ES IT	Basal leaves, stewed Basal rosettes, boiled in mixtures

## Data Availability

Not applicable.
